# Steigende Infektparameter nach Start der nasogastralen Sondennahrung

**DOI:** 10.1007/s00104-025-02325-9

**Published:** 2025-06-24

**Authors:** Kilian Doßow, Steffi Peglow, Frank Benedix, Ahmed Afifi, Mohammad Abdallah Alhabahbeh, Christine March, Roland Croner, Frank Meyer

**Affiliations:** 1 Klinik für Allgemein‑, Viszeral‑, Gefäß- und Transplantationschirurgie, Universitätsklinikum Magdeburg A. ö. R., Leipziger Str. 44, 39120 Magdeburg, Deutschland; 2 Klinik für Gastroenterologie, Hepatologie und Infektiologie, Universitätsklinikum Magdeburg A. ö .R., Magdeburg, Deutschland; 3 Klinik für Hals‑, Nasen- und Ohrenheilkunde, Kopf- und Halschirurgie, Universitätsklinikum Magdeburg A. ö .R., Magdeburg, Deutschland; 4 Klinik für Radiologie und Nuklearmedizin, Universitätsklinikum Magdeburg A. ö .R., Magdeburg, Deutschland

## Kasuistik

### Anamnese

Ein männlicher 72-jähriger Patient erhielt eine Aneurysmaausschaltung durch eine Bifurkationsprothesenimplantation als aortobifemoralen Bypass mittels 18 × 9 mm durchmessender Gelsoft™Plus-Prothese (Vascutek Deutschland GmbH, Hamburg, Deutschland) bei einem symptomatischen, gedeckt perforierten Bauchaortenaneurysma (BAA) von ca. 6,5 cm Durchmesser.

Im *Nebendiagnoseprofil* bot der Patient präoperativ einen arteriellen Hypertonus, eine chronische Niereninsuffizienz mit subtotaler Nierenarterienabgangsstenose rechts durch das bestehende BAA, eine Spinalkanalstenose mit chronischem Schmerzsyndrom, eine Arteriosklerose und einen aktiven Nikotinabusus.

Postoperativ traten nach initialer Kreislaufstabilität ohne Katecholamintherapie am 2. postoperativen Tag (POD) progrediente Unruhe im Sinne eines Delirs sowie eine Dyspnoe mit zunehmender respiratorischer Insuffizienz und Sauerstoffmangel auf.

Computertomographisch (CT) wurden Lungenarterienembolie und Pneumothorax ausgeschlossen mit Verdachtsdiagnose einer beginnenden Pneumonie bei bildgebend dargestellten peribronchialen Infiltraten, der mit einer kalkulierten Antibiotikatherapie (Piperacillin/Tazobactam; 3‑mal 4,5 g i.v.; Fresenius KABI GmbH, Bad Homburg vor der Höhe, Deutschland) gerecht wurde. Zudem wurde ein NSTEMI (Nicht-ST-Strecken-Elevationsmyokardinfarkt) laborchemisch und echokardiographisch diagnostiziert. Es erfolgte eine Herzkatheteruntersuchung mit Diagnosestellung einer relevanten 3-Gefäß-koronaren Herzkrankheit (KHK). In der hiesigen „interdisziplinären Herzkonferenz“ wurde die Indikation zur Hybridversorgung im frühelektiven Setting gestellt.

**Abb. 1:**
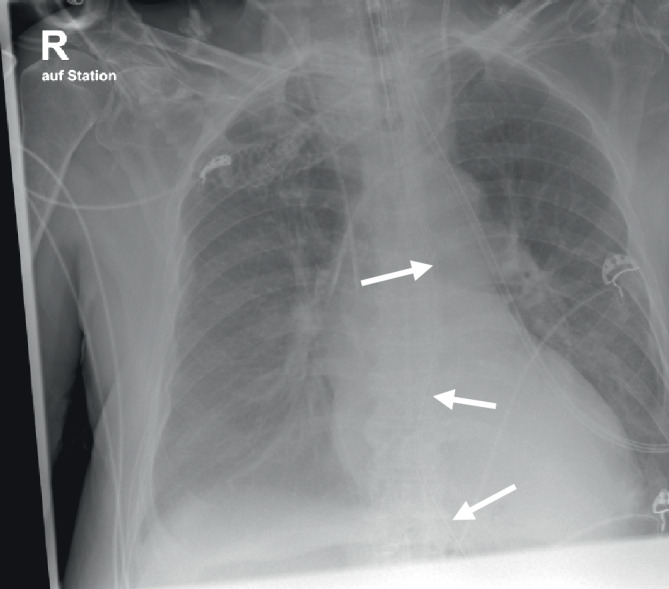
Konventionelles a.-p. Röntgenbild des Thorax im Liegen: Neben den korrekt liegenden zentralen Venenkathetern zeigt sich der Röntgenstreifen der NGT (*Pfeile*) mutmaßlich korrekt im Ösophagus. (Abbildungsquelle: Aus dem klinischen Image- und Bilddokumentenfokus der Klinik für Radiologie und Nuklearmedizin; Otto-von-Guericke-Universität mit Universitätsklinikum, Magdeburg, Deutschland)

Bei pulmonaler Verschlechterung musste der Patient am 7. POD reintubiert werden. Zur frühzeitigen enteralen Zusatzernährung wurde eine nasogastrale Ernährungssonde („nasogastric tube“ [NGT]) angelegt, die laut klinikinterner Dokumentation ein erschwertes Vorschieben, jedoch eine positive epigastrische Luftprobe auskultatorisch bot. In der Thoraxröntgenaufnahme zur Lagekontrolle eines zentralen Venenkatheters projizierte die NGT eine mutmaßlich korrekte Lage im Ösophagus. Die Spitze im Magen war nicht im Thoraxausschnitt abgebildet (Abb. 1). Die Verabreichung der Sondennahrung wurde nach initialem Versuch mit Tee in langsamer Flussgeschwindigkeit (10 ml/h) etabliert.

### Problemkonstellation

Nach Steigerung der kontinuierlichen Sondenkost fielen am 2. Tag nach Anlage deutlich steigende Infektparameter auf.

## Wie lautet Ihre Diagnose?

### Diagnostik

**Abb. 2:**
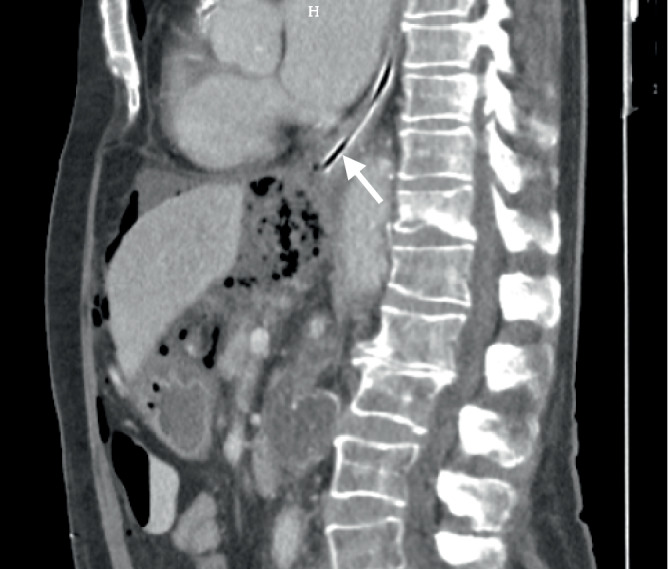
CT-Aufnahme in Sagittalebene: Es zeigen sich das beschriebene Luft-Flüssigkeits-Gemisch retrohepatisch sowie wenig freie Luft intraabdominell. Auf Zwerchfellebene scheint die NGT korrekt im Ösophagus zu liegen (*Pfeil*). (Abbildungsquelle: Aus dem klinischen Image- und Bilddokumentenfokus der Klinik für Radiologie und Nuklearmedizin; Otto-von-Guericke-Universität mit Universitätsklinikum, Magdeburg, Deutschland)

Es erfolgte eine *CT des Abdomens* zur Fokussuche. Hier zeigte sich eine extragastral liegende Magensondenspitze mit umgebendem Luft-Flüssigkeits-Gemisch im Oberbauch (Abb. 2 und 3).

**Abb. 3:**
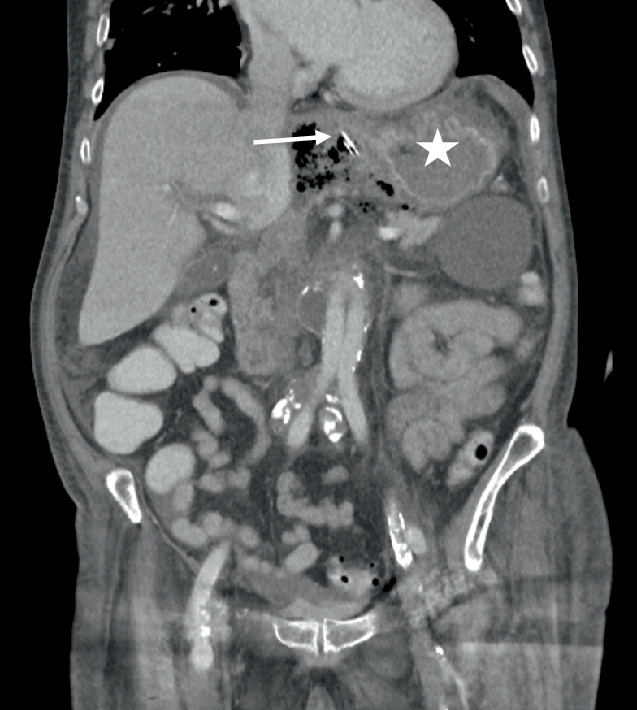
CT-morphologische Darstellung der fehlliegenden NGT (*Pfeil*) mit umgebender Sondenflüssigkeit und Lufteinschlüssen neben dem Magen (*Stern*). (Abbildungsquelle: Aus dem klinischen Image- und Bilddokumentenfokus der Klinik für Radiologie und Nuklearmedizin; Otto-von-Guericke-Universität mit Universitätsklinikum, Magdeburg, Deutschland)

**Abb. 4:**
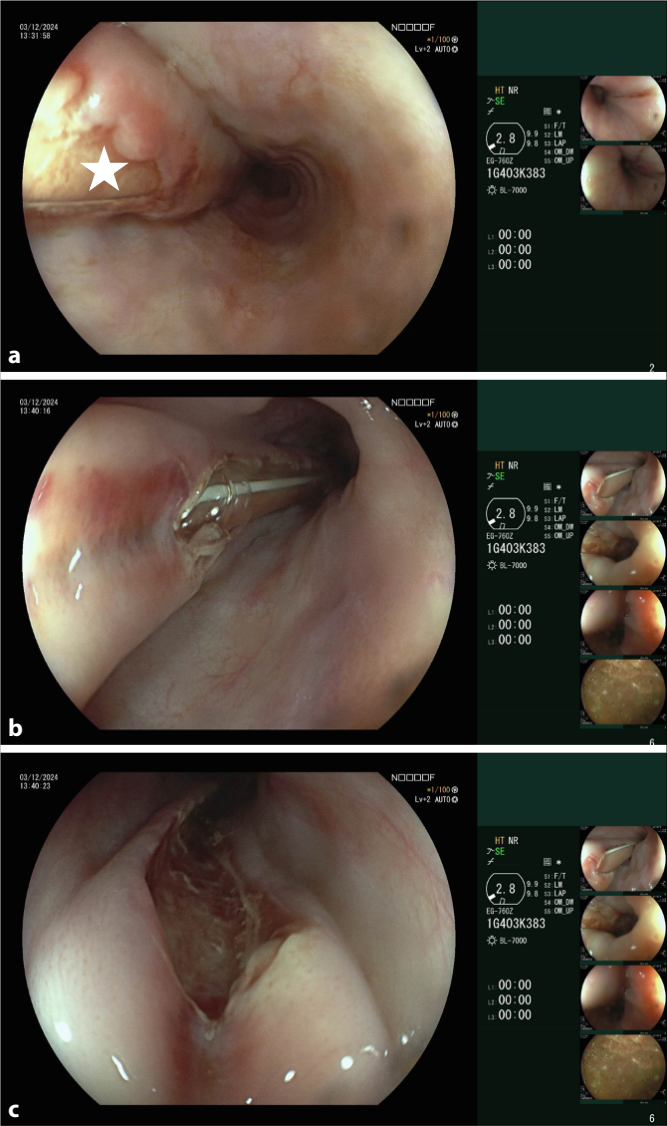
**a** Endoskopische Darstellung der unter der Ösophagusmukosa liegenden NGT (*Stern*). **b** Endoskopische Darstellung der fehlliegenden NGT mit segmentalem Durchbruch durch die Ösophagusmukosa. **c** Mukosaläsion nach Entfernung der fehlliegenden NGT. (Abbildungsquelle: Aus dem klinischen Image- und Bilddokumentenfokus der Klinik für Gastroenterologie, Hepatologie und Infektiologie, Abteilung Endoskopie; Otto-von-Guericke-Universität mit Universitätsklinikum, Magdeburg, Deutschland)

Die *Ösophagogastroskopie *bestätige die Fehllage der Ernährungssonde submukosal entlang des gesamten Ösophagus durch das endoskopisch eruierbare Durchscheinen der NGT (Abb. 4a, b). Intragastral konnte weder die Magensondenspitze noch eine Perforationsstelle eruiert werden.

## Dringende Verdachtsdiagnose

### Therapie

**Abb. 5:**
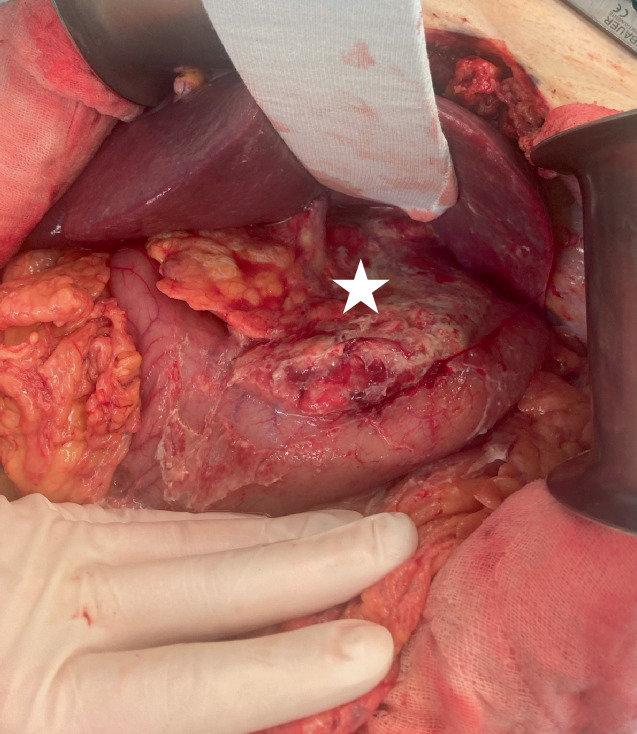
Intraoperativer Blick: verdicktes Omentum minus (*Stern*) in einem sonst weitgehend unauffälligen Situs. Auch unter Luft- und Betabraunol-Mischungs-Test keine Darstellung einer offenen Läsion. (Abbildungsquelle: Aus dem klinischen Image- und Bilddokumentenfokus der Klinik für Allgemein‑, Viszeral‑, Gefäß- und Transplantationschirurgie; Otto-von-Guericke-Universität mit Universitätsklinikum, Magdeburg, Deutschland)

Die fehlliegende Sonde wurde endoskopisch entfernt (Abb. 4c) und die Indikation zur notfallmäßigen Exploration gestellt unter Anlage einer neuen NGT in korrekter Lage. Die notfallmäßige Relaparotomie über den bestehenden Medianschnitt konnte intraoperativ keine freie Perforation oder frei liegende Sondennahrung im Abdomen darstellen. Weder am Magen noch im Bereich des Hiatus zeigte sich ein Serosa- oder Peritonealdefekt. Ein deutlich verhärtetes Gewebe an der kleinen Magenkurvatur ließ eine inkomplette Perforation mit Sondennahrungsapplikation (intramural) in die Magenwand oder das Omentum minus vermuten (Abb. 5). Wir legten nach ausgiebiger Lavage 3 abdominelle Robinson-Drainagen mit Option zur Spülung an.

### Postviszeralchirurgisches Procedere

Intra- und postoperativ konnte durch die Kollegen der Klinik für Hals‑, Nasen‑, Ohrenheilkunde (HNO) weder in der *Laryngoskopie *noch in der *flexiblen nasopharyngealen Endoskopie *die Eintrittsmukosaperforation gefunden werden.

### Weiterer Verlauf

Der weitere postoperative Verlauf gestaltete sich mit einer nur mäßigen pulmologischen Rekonvaleszenz und Etablierung einer Nierenersatztherapie letztendlich letal aufgrund der Ablehnung einer Tracheotomie und folgenden Bypassoperation am Herzen durch die Angehörigen als Ausdruck des mutmaßlichen Willens des Patienten.

## Diskussion

Der Ernährung kritisch kranker Patienten auf der Intensivstation kommt eine hohe Bedeutung zu. Neben der Vermeidung bzw. Minderung einer zügigen mukosalen und Zottenatrophie bei nicht gegebener enteraler Nutrition senkt eine frühzeitige Zuführung hochkalorischer Nahrung die Mortalität und Intensivtherapiedauer [23]. Der einfachste und schnellste Weg zur Etablierung einer enteralen Ernährung und damit zur Funktionserhaltung des Gastrointestinal(GI)-Trakts ist die Einlage einer NGT [5, 11, 18]. Die enterale Ernährung ist weniger invasiv und erlaubt die physiologische Absorption der Nährstoffe sowie die Anregung einer regelrechten Magen-Darm-Funktion [10].

Die Indikation zur NGT besteht zudem bei Patienten mit vorübergehender Schluckstörung [6] sowie zur frühzeitigen enteralen Ernährung bei Patienten mit Langzeitbeatmung oder prolongiertem postoperativem Weaning. Die Implantation einer perkutanen endoskopischen Gastrostomie (PEG) kann ebenfalls eine enterale Ernährung sichern, ist jedoch ein invasives Verfahren mit entsprechenden Risiken und weniger geeignet für die kurz-temporäre Notwendigkeit der Nutrition [17].

Die frühzeitige Ernährung von auf ITS hospitalisierten Patienten ist mitentscheidend für die Mortalität und Länge des Aufenthaltes im Intensivbereich [23]. Die NGT ist der schnellste und einfachste Weg zur Etablierung einer enteralen Ernährung [5] und ist aus dem klinischen Alltag der Intensivmedizin nicht wegzudenken.

Eine weitere Verwendung von Sonden über den nasogastralen Zugangsweg besteht in der Ersttherapie zur Magendekompression, insbesondere bei gastrointestinalen oder viszeralchirurgischen/-medizinischen Erkrankungen wie der Ileuskrankheit. Bei korrekter Lage kann diese drucklindernd und zustandsverbessernd wirksam werden sowie insbesondere lebensrettend vor Regurgitation, Erbrechen und Aspiration mit konsekutiver Pneumonie schützen [10].

Trotz der geringen Invasivität der NGT-Anlage, verglichen mit perkutanen endoskopischen oder operativen Zugängen und der damit positiven Nutzen-Risiko-Abwägung in den oben genannten medizinischen Szenarien, verbleibt ein nicht zu vernachlässigendes Komplikationsprofil, wenn auch insgesamt rar, jedoch mit breit gefächertem Verletzungsmuster und unterschiedlichsten Folgen. Dieses reicht vom Larynxödem über bronchopulmonale Komplikationen bei Fehlintubation der Trachea [16] bis zur Perforation der Ösophagus- und Magenwand [1, 7] mit teilweise letalen Folgen [14].

Abhängig von der Liegedauer kann der einliegende Fremdkörper zu Schleimhautreizungen, -erosionen und Infektionen führen. Eder et al. beschreiben eine entzündliche Ösophagusstenose durch eine intraoperativ eingelegte NGT [3]. Laryngeal zeigt sich durch Druckschäden des Fremdkörpers bei bis zu einem Drittel der Patienten mit NGT ein Aryknorpelödem [20], teilweise mit konsekutiver laryngealer Schleimhautinfektion und passagerer Stimmlippenparalyse, dem sog. „nasogastric tube syndrome“ (NGTS) [22].

Größtes Augenmerk bei der Anlage der NGT liegt auf der korrekten Intubation des Ösophagus. Die Fehleinlage in die Trachea und das Bronchialsystem kann zu einer Reihe von teilweise schwerwiegenden Verletzungen des bronchopulmonalen Organsystems führen: Neben Blutungen und oberflächlichen Verletzungen des Flimmerepithels kann es durch die stetige Verkleinerung des Lumendurchmessers beim Vorschub der NGT leicht zu Perforationen der Bronchien kommen. Folgen können sein: Pneumonie, Pneumothorax und Pleuraempyem [8, 12]. Bei Beginn einer Sondenbestückung der fehlliegenden Magensonde führt dies zur Fehlapplikation in den/das nicht-gastrointestinalen Raum/Lumen. Ebenfalls sind seltene Fehllagen intrakranial oder im Hirnstamm beschrieben [6].

Gelingt die korrekte Intubation der NGT am Larynx vorbei in den Ösophagus, ist ein Auftreten von Komplikationen wie Verletzung der Magen- oder Ösophaguswand dennoch möglich [7, 14]. Insbesondere die Ösophagusperforation, die in mehr als der Hälfte aller Fälle iatrogen verursacht wird [26], stellt mit der Gefahr einer Mediastinitis eine lebensbedrohliche Situation dar. Insbesondere unter dem Augenmerk der NGT-Einlage ohne Sicht [18] handelt es sich um eine Komplikation, die der versierte Notfall- und Intensivmediziner im Hinterkopf haben sollte.

Die Einlage einer NGT in Situationen der Notfall- und intensivmedizinischen Betreuung wird auch zukünftig ein weiterer fester Bestandteil der Therapie(maßnahmen) bleiben, insbesondere unter der Betrachtung des Risiko-Nutzen-Verhältnisses. Dennoch besteht die Frage nach einer Verminderung der anlagebedingten Komplikationen und dem optimalen Komplikationsmanagement.

Die Fehleinlage der NGT wird in der Literatur zwischen 1,3 und 2,4 % beschrieben [12, 21]. Die Technik der Einlage kann für ärztliches und pflegerisches Personal am Simulator geübt werden. Bei Auftreten von Widerständen sollte, insbesondere bei mehreren frustranen Versuchen, vom blinden Vorschieben abgesehen und ggf. eine laryngoskopische oder endoskopische Assistenz erwogen werden [11].

Der *Lagekontrolle* kommt nicht zuletzt bei geplanter Sondenernährung und möglicher Fehlapplikation in anatomische und extraanatomische Räume eine große Bedeutung zu.

Die Luftprobe durch Insufflation von Luft über die Magensonde mit positivem Flush-Geräusch in der epigastrischen Auskultation mit dem Stethoskop ist einfach und schnell sowie unverzüglich und kostensparend durchzuführen. Eine eindeutige Sicherheit zur korrekten Lage besteht jedoch nicht [25].

Additiv kann und sollte dies durch eine pH-Messung des NGT-Aspirates (pH 4) [4] oder bildgebende Verfahren erweitert werden. Die Röntgenuntersuchung kann eine Fehllokalisation, zumindest was die Fehlintubation des Bronchialsystems angeht, eindeutig ausschließen, bedeutet aber einen Zeitverzug, erhöhte Kosten und eine Strahlenbelastung [18]. Zudem ist sie nicht in jeder Situation zeitnah vorhanden bzw. unmittelbar durchführbar.

Abhängig vom Untersucher und Situs ist es möglich, eine NGT-Lokalisation mittels Ultraschalldiagnostik ohne Zeitverzug intragastral oder als doppelte Sicherung zusätzlich intraösophageal über einen Halsultraschall nachzuweisen [2]. Eine zusätzliche Applikation („Flush“) von Flüssigkeit kann notwendig sein, um eine eindeutige Aussage treffen zu können. In der Aussage über die Lokalisation einer fehlgelegten NGT ist der Ultraschall dem Röntgen unterlegen [24].

Die Einlage unter endoskopischer Kontrolle sichert die korrekte Ösophagusintubation in Echtzeit, ist im klinischen Alltag sicher jedoch kaum in jedem Fall realisierbar. Insbesondere mit der Indikation der Dekompression bei Magenausgangsstörungen und der Ileuskrankheit sollte keine Zeitverzögerung stattfinden. Alternativ kann – zumindest beim beatmeten Intensivpatienten – eine NGT-Einlage unter Sicht mithilfe eines Laryngoskops oder GlideScopes durchgeführt werden [18].

Im vorgetragenen Fall erfolgte eine Röntgenaufnahme des Thorax zur Lagekontrolle eines neuen zentralen Venenkatheters, die gleichzeitig eine mutmaßlich korrekte Lage der NGT im Ösophagus zeigte. Die vermutete intragastrale Lokalisation der NGT-Spitze war in der Thoraxaufnahme nicht mit abgebildet (Abb. 1). Wenn auch nicht Teil der Fragestellung der Thoraxaufnahme, wurde in dieser Röntgenaufnahme durch den langstreckigen Verlauf der NGT in der Ösophaguswand fälschlicherweise eine korrekte Lage interpretiert.

Das *Management* nach NGT-Komplikation richtet sich je nach vorhandener Verletzung bzw. Lokalisation. Im Falle einer Ösophagusverletzung mit Perforation steht die Zeit bis zur Therapie im direkten Zusammenhang mit der Mortalität und steigt von 10–25 % bei Therapieeinleitung innerhalb von 24 h auf 40–60 % bei Therapieverzug über 48 h [9].

Eine unverzügliche radiologische Diagnostik mittels Röntgen oder CT sichert eine weiterführende Therapieentscheidung. Im beschriebenen Fall erfolgte eine CT-Diagnostik des Abdomens bei unklarem Infektanstieg, welche die fehlliegende NGT-Spitze mit perigastraler Flüssigkeit und Luft zeigte (Abb. 3). Zuvor war die Sondenkost per Infusomat gestartet worden, über deren Funktion keine Störung dokumentiert wurde.

Eine Endoskopie zur Detektion der Dislokationsstelle erfolgte unverzüglich nach CT-Diagnostik. Hier zeigte sich der langstreckige Verlauf der NGT submukosal im Ösophagus ohne Anhalt auf die Spitzendislokationsstelle im Bereich des Magens.

Die Endoskopie selbst kann im Falle von iatrogenen Perforationen eine Therapieoption bieten. Die Verwendung von „Over-the-scope“-Clips (OTSC) oder „Through-the-scope“-Clips (TTSC) können in individuellen Fällen eine zügige und weniger invasive Alternative zur operativen Versorgung darstellen und gastrointestinale Läsionen und Perforationen in 60–100 % verschließen [13, 15, 19]. Nichtsdestotrotz sollte eine frühzeitige chirurgische Konsultation zur interdisziplinären Therapieentscheidung erfolgen.

Im präsentierten Falle konnte die submukosale Lage im Ösophagus endoskopisch durch das Durchscheinen der NGT gesichert werden, im Magen war eine endoskopische Versorgung bei fehlender darstellbarer Austrittsstelle jedoch nicht möglich und eine freie Perforation in die Peritonealhöhle im CT nicht auszuschließen.

Eine chirurgische Exploration war somit indiziert, ein laparoskopischer Versorgungsversuch wurde bei kurzfristigem Zustand nach offener BAA-Versorgung mittels Prothese nicht erwogen. Bei makroskopisch sowie auch in der Luftdichtheitsprobe des Magens fehlender freier Perforation waren die Lavage und Anlage mehrerer Drainagen mit postoperativer Spüloption die Therapie der Wahl vor Ort im intraoperativen Situs. In der Kontrolle durch den konsultierten Arzt aus der Klinik für HNO ließ sich der Defekt der Mukosaeintrittsstelle der NGT weder in der Laryngoskopie noch in der flexiblen Nasopharyngoendoskopie sichern.

Der vorgestellte Patient erlag trotz zügiger, individuell an die Verletzung angepasster Therapie seinen pulmonalen und interventionsbedürftigen kardialen Komorbiditäten.

**Diagnose:** Submukosale langstreckige Ösophagusdissektion durch die Anlage einer NGT

Als Anmerkung an das eigene, einrichtungsinterne Komplikationsmanagement bleibt:

Das Belassen der fehlliegenden NGT zur flexiblen Laryngoendoskopie und explorativen Laparotomie hätte besseren Aufschluss über den genauen Verlauf des Fremdkörpers sowohl bei Eintritt in die Mukosa als auch bei Austritt ins Omentum minus geben können. Ob sich dadurch eine Änderung des Therapiekonzepts und des Outcomes ergeben hätte, bleibt jedoch spekulativ.

## Fazit für die Praxis

- Die Dekompression des Magens zum Schutz vor Aspiration sowie die Notwendigkeit einer frühzeitigen (enteralen) Ernährung beim kritisch kranken Patienten stellen die Indikation für die rechtzeitige Etablierung einer NGT dar.
- Die Anlage einer NGT entspricht dem einfachsten und schnellsten Weg zur Magendekomprimierung oder enteralen Ernährung.
- Das Risiko- und Komplikationsprofil sollten insbesondere bei der blinden Anlage einer NGT berücksichtigt werden. Bei erschwertem Zugang empfiehl sich ggf. eine endoskopische Assistenz.
- Die Lagekontrolle einer NGT sollte mindestens mit Luftprobe in der Auskultation, präferabel kombiniert mit einer bildgebenden (Röntgen, Ultraschall) oder pH-metrischen Kontrolle erfolgen.
- Das Komplikationsmanagement richtet sich nach dem jeweiligen Verletzungsmuster durch die NGT. Ein Zeitverzug bis zur Diagnostik und Therapie steht in direktem Zusammenhang zur Mortalität.
